# Evidence for cohesin sliding along budding yeast chromosomes

**DOI:** 10.1098/rsob.150178

**Published:** 2016-06-08

**Authors:** Maria Ocampo-Hafalla, Sofía Muñoz, Catarina P. Samora, Frank Uhlmann

**Affiliations:** The Francis Crick Institute, Lincoln's Inn Fields Laboratory, 44 Lincoln's Inn Fields, London WC2A 3LY, UK

**Keywords:** cohesin, genome stability, *Saccharomyces cerevisiae*, sister chromatid cohesion, transcription

## Abstract

The ring-shaped cohesin complex is thought to topologically hold sister chromatids together from their synthesis in S phase until chromosome segregation in mitosis. How cohesin stably binds to chromosomes for extended periods, without impeding other chromosomal processes that also require access to the DNA, is poorly understood. Budding yeast cohesin is loaded onto DNA by the Scc2–Scc4 cohesin loader at centromeres and promoters of active genes, from where cohesin translocates to more permanent places of residence at transcription termination sites. Here we show that, at the *GAL2* and *MET17* loci, pre-existing cohesin is pushed downstream along the DNA in response to transcriptional gene activation, apparently without need for intermittent dissociation or reloading. We observe translocation intermediates and find that the distribution of most chromosomal cohesin is shaped by transcription. Our observations support a model in which cohesin is able to slide laterally along chromosomes while maintaining topological contact with DNA. In this way, stable cohesin binding to DNA and enduring sister chromatid cohesion become compatible with simultaneous underlying chromosomal activities, including but maybe not limited to transcription.

## 1. Introduction

Cohesin is a ring-shaped multi-subunit protein complex, which is best known for its essential role in promoting sister chromatid cohesion [1–4]. The cohesin ring topologically encircles DNA and, in mitosis, cohesin entraps and thereby holds together pairs of sister chromatids. The cohesin complex consists of at least four essential subunits. At the core of the complex lies a dimer of structural maintenance of chromosomes (SMC) ATPases, Smc1 and Smc3. These form the ring via interaction interfaces at both ends of long flexible stretches of coiled-coil, an SMC hinge and the ATPase heads [5,6]. Two additional subunits of the complex are essential in all organisms studied, called Scc1 and Scc3 in budding yeast. Scc1 stabilizes the interaction between the SMC ATPase domains. Scc3 in turn associates with Scc1 and plays an essential role in loading of the cohesin complex onto DNA [7,8]. It does so by providing interactions with a separate cohesin loader complex that in budding yeast consists of the Scc2 and Scc4 subunits [9]. In addition to this cohesin core machinery, additional subunits Pds5, Wapl and sororin, the latter restricted to higher eukaryotes, associate with the cohesin complex to regulate the stability of cohesin binding to chromosomes.

A crucial moment for cohesin comes at the time of DNA replication. Two newly replicated sister chromatids emerge from the replication fork, which cohesin will hold together until mitosis. To ensure stable sister chromatid cohesion from S phase until mitosis, the cohesin ring is modified during DNA replication. The replication fork-associated acetyltransferase Eco1 acetylates cohesin rings on their Smc3 subunit, which stabilizes cohesin's grip on chromosomes [10–12]. This is achieved in at least two ways. First, acetylation renders cohesin resistant to a DNA unloading activity contained in the Wapl subunit. Dynamic cohesin unloading by Wapl is important to fine-tune the chromosome condensation status in both interphase and mitosis, but during DNA replication a subset of cohesin is rendered Wapl-resistant by Eco1 [13–16]. In addition, acetylation stabilizes cohesin on DNA by a second mechanism [17]. An explanation for both mechanisms has recently been suggested by the finding that the acetylation sites form a DNA sensor that triggers ATP hydrolysis, required for DNA entry into and exit out of the cohesin ring [18]. As the consequence, acetylated cohesin gains close to permanent association with chromosomes.

In budding yeast, acetylated cohesin remains chromosome-bound for longer than could be directly measured, at least for an hour [14]. In human cells, following cohesion establishment during DNA replication and assisted by the additional sororin subunit [19], about a third of cohesin complexes are similarly close to permanently stabilized and turn over on chromosomes with a rate that was again slower than could be measured, remaining chromosome-bound for at least 6 h [13]. An argument has been made that, in mammalian female oocytes, cohesin rings might persist on chromosomes for weeks (if not decades) to maintain sister chromatid cohesion in meiotic prophase arrested cells [20,21].

While cohesin is stably bound to chromosomes to provide sister chromatid cohesion, other forms of chromosomal metabolism must continue unabated underneath. Much of the budding yeast genome consists of genes that are frequently transcribed. Almost the entirety of the human genome is also transcribed, at least occasionally [22,23]. In addition, numerous DNA lesions continuously accrue and must be repaired in every cell, involving repair pathways that require access to the DNA. This includes for example nucleotide excision repair or DNA recombination events, both of which require DNA resynthesis to proceed over substantial distances. How cohesin can maintain stable sister chromatid cohesion over long periods of time, without restricting access to genomic regions for transcription, repair and other chromosomal requirements is not known.

We have previously observed that transcriptional activation of budding yeast genes can lead to a striking repositioning of cohesin along chromosomes, with cohesin apparently moving downstream of active genes to accumulate at convergent transcriptional termination sites [24]. Lateral sliding of cohesin, while retaining topological contact, has been suggested as a possible explanation for how the genome stays accessible while cohesin remains bound [25]. However, the actual mechanism of cohesin translocation is not known. In particular, it is not known whether relocation in response to transcriptional activation occurs while cohesin remains in contact with DNA or whether it requires cohesin dissociation from DNA and subsequent reloading at a new location. In the latter scenario, sister chromatid cohesion would gradually deteriorate during cohesin translocation, as under normal growth conditions cohesin can establish sister chromatid cohesion only during S phase, but not afterwards [12,26]. Indeed, a previous study that analysed cohesin's response to changes in the transcriptional programme concluded that cohesin is displaced from chromosomes in response to transcription [27]. How a transcription-mediated decay of sister chromatid cohesion over time is prevented in this scenario has not been explained.

Given the importance of stable sister chromatid cohesion, we have revisited the nature of cohesin translocation along budding yeast chromosome arms in response to transcriptional changes. We study two model loci where transcriptional induction leads to downstream translocation of cohesin. Cohesin behaviour is observed by chromatin immunoprecipitation (ChIP) followed by analysis on high-resolution oligonucleotide tiling arrays. We find that, following gene activation, cohesin can move downstream of genes without requirement for known dissociation or reloading factors and we observe a translocation intermediate, consistent with cohesin sliding along genes while retaining topological association. At another locus, close to a centromere, transcriptional activation leads to the apparent loss of cohesin from chromosomes. Together, our results support a model in which, at least along chromosome arms, cohesin retains topological contact with DNA and thereby sister chromatid cohesion, while sliding laterally to accommodate requirements of transcription and probably other forms of chromosomal metabolism.

## 2. Results

### 2.1. Cohesin translocation without the cohesin loader

**Figure 1.**
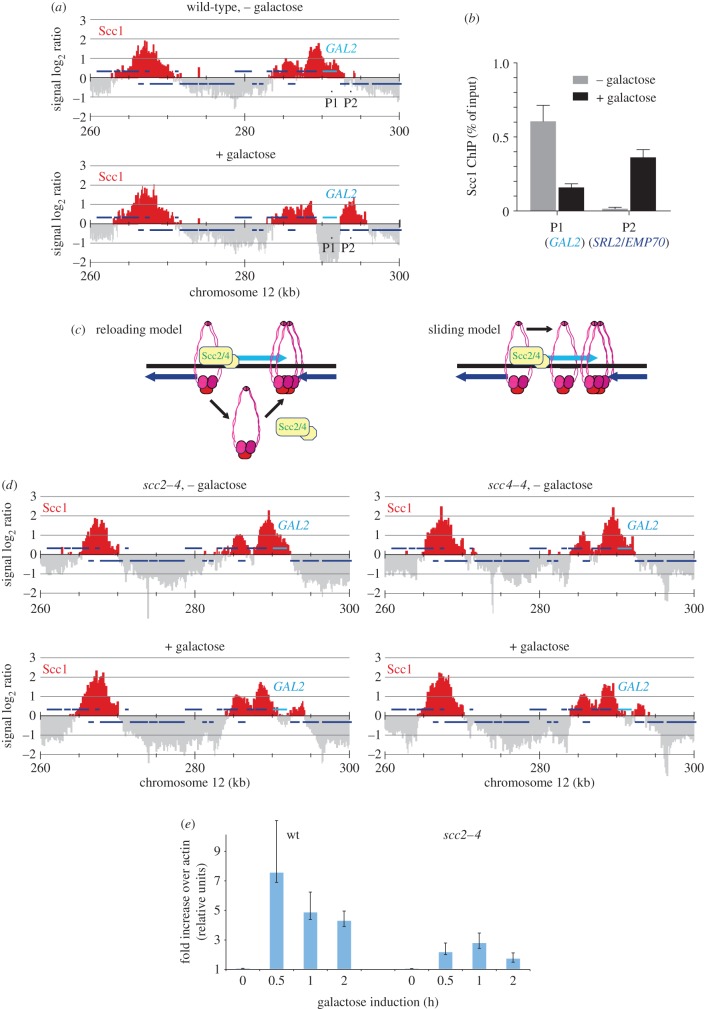
Contribution of Scc2–Scc4 to cohesin translocation at the *GAL2* locus. (*a*) Cohesin translocation following *GAL2* induction. Cells were grown in raffinose-containing medium, synchronized in G1 and released into nocodazole-induced G2/M arrest. After 2 h, the culture was shifted to 35°C and after a further hour galactose was added to induce *GAL2* expression. Scc1 association with chromatin was analysed before (− galactose) and one hour following induction (+ galactose). Enrichment in the immunoprecipitate relative to a whole genome DNA sample is shown along a region surrounding the *GAL2* locus. Each bar represents the average of 25 oligonucleotide probes within adjacent 125 bp windows. Blue bars above and below the midline are genes transcribed, respectively, from left to right, and from right to left. The location of two primer pairs, P1 and P2, used for analysis of chromatin immunoprecipitates by quantitative real-time PCR in (*b*), are indicated. (*b*) Analysis of Scc1 chromatin immunoprecipitates using primer pairs P1 and P2 by quantitative real-time PCR, before and 1 h after galactose addition. The means and standard error of three repeats of the experiment are shown. (*c*) Schematic of cohesin translocation by either reloading or sliding. Indicated is the predicted requirement for the Scc2–Scc4 cohesin loader in the former, but not the latter, case. (*d*) Cohesin translocates at a reduced rate following inactivation of the Scc2–Scc4 cohesin loader. As (*a*), but cells carried temperature-sensitive alleles of the cohesin loader subunits. (*e*) Compromised *GAL2* induction following cohesin loader inactivation. *GAL2* mRNA induction following galactose addition, relative to actin, was assessed using quantitative real-time PCR in both a wild-type and *scc2–4* strain. The means and standard deviation from two biological and four technical replicates are shown.

As a model locus to study cohesin translocation, we chose the *GAL2* locus on the right arm of budding yeast chromosome 12 [27,28]. There, as an exception to the typical location of cohesin peaks at convergent intergenic regions, three sequential genes in tandem orientation, *SIC1*, *EMP46* and *GAL2* are covered by cohesin. This is the case in cells grown in medium containing raffinose as the carbon source and arrested in G2/M by nocodazole treatment, when the three genes are barely transcribed (figure 1*a*) [29]. Following addition of galactose to the culture, which induces *GAL2* expression, the *GAL2* gene is cleared of cohesin. Instead, in an aliquot of the same culture taken 1 h after galactose addition, a marked cohesin peak appears downstream of *GAL2* (figure 1*a*). The remainder of the genomic cohesin distribution remained largely unchanged following galactose addition, allowing the relative quantitative comparison of cohesin at the *GAL2* locus with the rest of the genome. An upstream cohesin binding region that remained unchanged is therefore included in our graphs for comparison. In addition, we analysed cohesin chromatin immunoprecipitates by quantitative real-time PCR using primer pairs both within the *GAL2* gene and downstream in the *SRL2*/*EMP70* intergenic region, before and after galactose addition. This confirmed the quantitative downstream relocation of cohesin from the *GAL2* locus (figure 1*b*).

The following experiments were designed to explore two possible explanations for cohesin translocation. It could be that cohesin is removed from chromosomes following transcriptional activation of the *GAL2* locus and a new cohesin binding site is established downstream, where cohesin is newly loaded. Alternatively, cohesin that topologically encircles DNA could be pushed downstream along chromosomes, without dissociation and therefore without need for reloading (figure 1*c*). To distinguish between these two possibilities, we first asked whether the Scc2–Scc4 cohesin loader is required for cohesin translocation. In the reloading model, this is likely to be the case. We therefore repeated the *GAL2* induction experiment in strains in which the cohesin loader can be inactivated owing to a temperature-sensitive *scc2–4* or *scc4–4* mutation [9]. Once cells were arrested in G2/M, they were shifted to 35°C for 1 h, a restrictive temperature for the *scc2–4* and *scc4–4* alleles, before galactose addition. Following either Scc2 or Scc4 inactivation, *GAL2* induction still caused cohesin translocation, but the extent of both cohesin loss from the *GAL2* locus and its downstream accumulation were reduced (figure 1*d*). This suggests that, while not absolutely required, the cohesin loader facilitates cohesin repositioning at the *GAL2* locus.

The cohesin translocation defect at the *GAL2* locus, following Scc2–Scc4 inactivation, could be for two reasons. Scc2–Scc4-dependent cohesin loading might augment a less efficient, loading-independent translocation reaction. This scenario would explain the reduced downstream cohesin peak, but would not explain why clearance of the *GAL2* locus is also less efficient in the absence of the cohesin loader. Alternatively, Scc2–Scc4 inactivation might hamper transcriptional activation of the *GAL2* locus, which could explain both reduced cohesin loss from the *GAL2* locus as well as reduced downstream accumulation. To investigate this possibility, we quantified *GAL2* induction by real-time PCR analysis of transcript levels. This revealed that *GAL2* induction was markedly reduced in *scc2–4* mutant cells, compared with the wild-type control (figure 1*e*). While we do not know how the cohesin loader promotes *GAL2* gene induction, we recently reported on its role in promoter nucleosome eviction, which might pertain to reduced gene activation [30]. Attenuated transcriptional activation, in turn, could explain the reduced efficiency of cohesin translocation. We conclude that cohesin translocation is possible without new cohesin loading. The mechanism by which Scc2–Scc4 augments the process at the *GAL2* locus remains to be further explored.

### 2.2. Loading-deficient cohesin is translocation proficient

**Figure 2.**
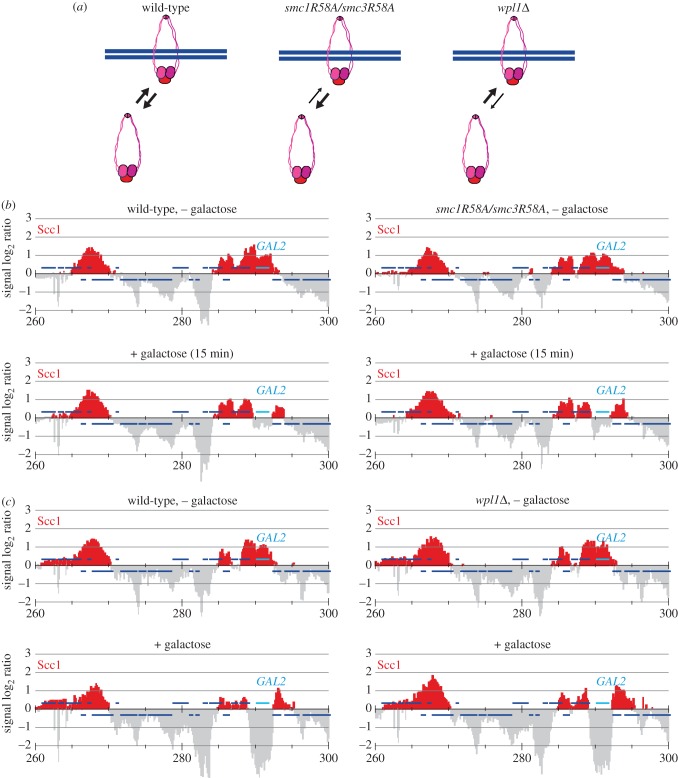
Altered cohesin dynamics do not impact on cohesin translocation. (*a*) Schematic of the respective consequences of the *smc1R58A/smc3R58A* and *wpl1Δ* mutations on dynamic cohesin association with chromosomes. (*b*) Cohesin translocation remains unaffected by the *smc1R58A/smc3R58A* mutations. Following release from α-factor arrest, cell cycle progression was halted for 2 h in HU-containing medium to allow cohesin accumulation on chromosomes, before cell cycle progression was allowed to resume and cells were arrested in G2/M by nocodazole treatment. Cohesin chromatin immunoprecipitation (ChIP) was then performed before and 15 min after *GAL2* induction by galactose addition. (*c*) Cohesin translocation remains unaffected in the absence of Wapl. Cohesin ChIP was performed in G2/M arrested cells before and 1 h after *GAL2* induction.

Given the complications with the interpretation of the above experiment, we used a complementary approach to test the contribution of new cohesin loading to transcription-induced translocation. Cohesin loading onto DNA requires hydrolysis of ATP, bound to the Smc1 and Smc3 head domains [8,31,32]. Mutation of an arginine finger in both ATPases (Smc1R58A/Smc3R58A) greatly reduces the rate of cohesin loading onto chromosomes. Starting from the G1/S transition, when the cohesin complex is assembled in budding yeast, it takes about 2 h longer for Smc1R58A/Smc3R58A cohesin to reach equal levels on chromosomes compared with wild-type cohesin [12] (figure 2*a*). If cohesin translocation involves ATP hydrolysis-dependent reloading, then downstream accumulation of Smc1R58A/Smc3R58A cohesin is expected to be reduced or delayed. To analyse whether this was the case, we performed the following experiment. Wild-type and Smc1R58A/Smc3R58A cells were synchronized in raffinose-containing medium by α-factor block and release. Cell cycle progression was halted by hydroxyurea (HU) treatment in early S phase for 2 h, to allow Smc1R58A/Smc3R58A cohesin to accumulate on chromosomes. Cultures were now released into nocodazole-containing medium, and the cohesin distribution in the G2/M-arrested cells was assessed at the *GAL2* locus before and after induction by galactose addition. Expression of budding yeast galactose-inducible genes becomes detectable around 8–12 min following galactose addition to the growth medium [33]. To sensitively detect a cohesin translocation difference due to compromised loading, we therefore took our second sample 15 min following galactose addition. In wild-type cells, cohesin translocation at the *GAL2* locus was largely complete by this time. Notably, translocation of Smc1R58A/Smc3R58A cohesin was similarly fast and efficient (figure 2*b*). Thus, while causing a marked delay in loading onto chromosomes, Smc1R58A/Smc3R58A cohesin was fully proficient in transcription-induced downstream relocation. This suggests that renewed ATP hydrolysis, and therefore renewed cohesin loading onto chromosomes, are unlikely to be part of the translocation mechanism.

### 2.3. Wapl-dependent unloading is dispensable for cohesin translocation

We took an additional approach to test whether a dissociation and reloading cycle forms part of cohesin translocation, by asking whether the dissociation activity of cohesin's Wapl subunit is involved in the process. Before DNA replication, cohesin associates with chromosomes in a dynamic fashion, undergoing cycles of loading and Wapl-mediated dissociation (figure 2*a*). Following DNA replication, acetylation turns cohesin resistant to Wapl, though Wapl retains an influence over at least a fraction of cohesin [15,16,18,30]. To test whether cohesin unloading from chromosomes by Wapl is involved in cohesin translocation, we compared a wild-type and a *wpl1Δ* strain, deleted for the gene encoding Wapl. Following synchronization, cells were again arrested in G2/M by nocodazole treatment and then *GAL2* gene expression was induced by galactose addition. Cohesin translocation occurred in an indistinguishable fashion in both wild-type and *wpl1Δ* cells (figure 2*c*). This observation again suggests that cohesin does not dissociate and reload during the translocation process.

### 2.4. Pre-existing cohesin relocates downstream

**Figure 3.**
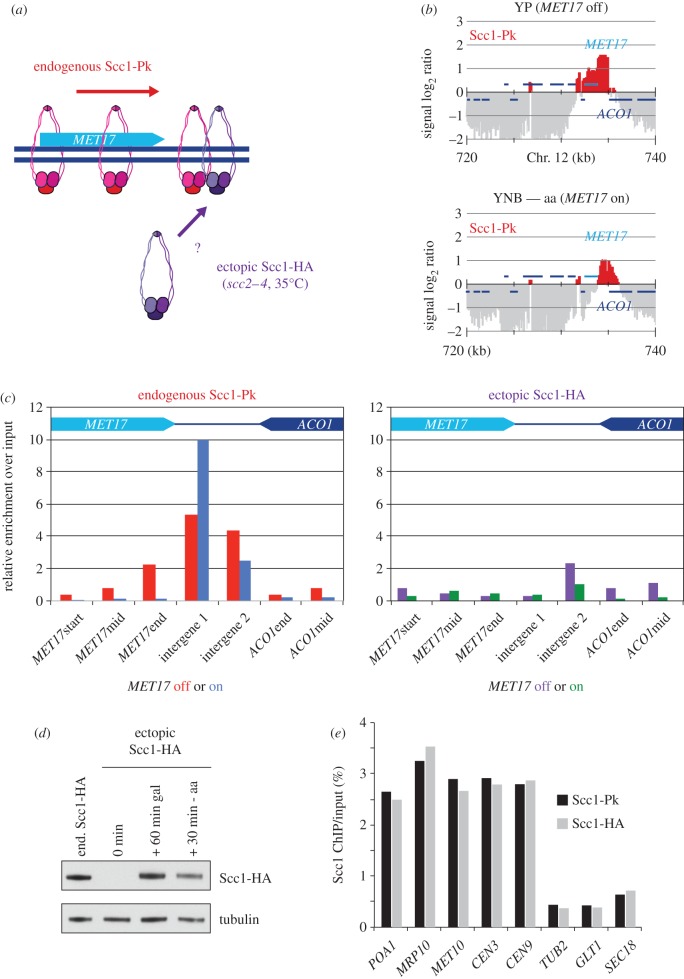
Pre-existing cohesin translocates at the *MET17* locus. (*a*) Schematic of the experiment to compare the behaviour of endogenous cohesin (containing Pk epitope-tagged Scc1) and ectopic cohesin (containing HA epitope-tagged Scc1) that was expressed after cohesin loader inactivation, but before cohesin translocation was induced. (*b*) Cohesin translocation at the *MET17* locus. Cohesin chromatin immunoprecipitation (ChIP) was performed in G2/M arrested cells, before (YP) and 30 min after transfer to medium lacking amino acids (YNB—aa). (*c*) Only pre-existing cohesin translocates. After 1.5 h following release from α-factor arrest into G2/M, cells were shifted to a restrictive temperature for the *scc2–4* allele for 1 h before Scc1-HA expression was induced by galactose addition for one further hour. Then cells were transferred to medium lacking amino acids. Cohesin ChIP was performed against both endogenous (Scc1-Pk) and ectopic (Scc1-HA) cohesin just before and 30 min following amino acids starvation. (*d*) Ectopic Scc1-HA levels at the indicated times were compared by immunoblotting to those in a control strain in which endogenous Scc1 was fused to the same HA epitope. Tubulin served as a loading control. (*e*) The efficiency of Scc1 chromatin immunoprecipitation using an HA and Pk epitope, respectively, was quantitatively compared at five cohesin binding sites and three negative control regions.

Another way to assess whether cohesin translocates by lateral movement, or by dissociation and reloading, is to determine the composition of the newly formed downstream cohesin pool. This pool could consist of previously chromosome-bound cohesin, as expected following lateral movement. Alternatively, it could include cohesin that was not previously chromosome-bound. To distinguish between these possibilities, we devised the following experiment. As before, cells containing the temperature-sensitive *scc2–4* allele were synchronized by α-factor block and release and arrested in G2/M by nocodazole treatment under permissive conditions. Endogenous cohesin, containing a Pk epitope-tagged Scc1 subunit for detection, is bound to chromosomes under these conditions. The cohesin loader was now inactivated by temperature shift. After this, we induced synthesis of an HA epitope-tagged Scc1 subunit, under control of the galactose-inducible *GAL1* promoter, for 1 h. The differentially epitope-tagged Scc1 assembles into functional new cohesin complexes [12], so that cells now contained two cohesin populations: endogenous Pk epitope-tagged cohesin that is bound to chromosomes, as well as HA epitope-tagged cohesin in the nucleoplasm (figure 3*a*). Now, we induced cohesin translocation and analysed the composition of the downstream cohesin pool.

As we used galactose to induce Scc1-HA synthesis, we used another translocation model locus, *MET17*, whose transcription is activated by amino acid starvation [27,28]. In rich medium, containing raffinose and galactose, endogenous Scc1-Pk containing cohesin covers the *MET17* gene, while the downstream, convergently transcribed *ACO1* gene is cohesin free (figure 3*b*). Thirty minutes after transfer to synthetic medium lacking amino acids, endogenous cohesin translocated downstream. *MET17* was now free of cohesin, whereas the *ACO1* locus was partly covered. This confirms our earlier results that cohesin translocation can occur independently of the Scc2–Scc4 cohesin loader. We next quantitatively analysed the composition of the new cohesin peak by ChIP followed by quantitative real-time PCR, using seven primer pairs across the *MET17*–*ACO1* locus. This analysis confirmed downstream translocation of endogenous Scc1-Pk-containing cohesin, whereas we did not detect HA epitope-tagged Scc1 from soluble cohesin above background levels at any of the locations, either before or after *MET17* induction (figure 3*c*). This suggests that the ‘new’ cohesin peak consists largely, if not exclusively, of previously chromosome-bound cohesin. The closest possible origin of cohesin in the new peak is those cohesin molecules that previously occupied the upstream location and appear to have moved downstream along the chromosome following transcriptional activation.

As a control for the above experiment, we confirmed that ectopic Scc1-HA, following 1 h of induction, reached levels comparable to those in a strain in which the endogenous *SCC1* locus is tagged with the same epitope (figure 3*d*). We furthermore confirmed that, under these conditions, both HA and Pk epitope-tagged cohesin is retrieved with similar efficiency by ChIP (figure 3*e*). This validates the quantitative comparison between ectopic and endogenous cohesin following *MET17* induction and supports the conclusion that little if any ectopic cohesin is incorporated into a newly established cohesin peak following transcriptional induction.

### 2.5. A translocation intermediate suggestive of cohesin sliding

**Figure 4.**
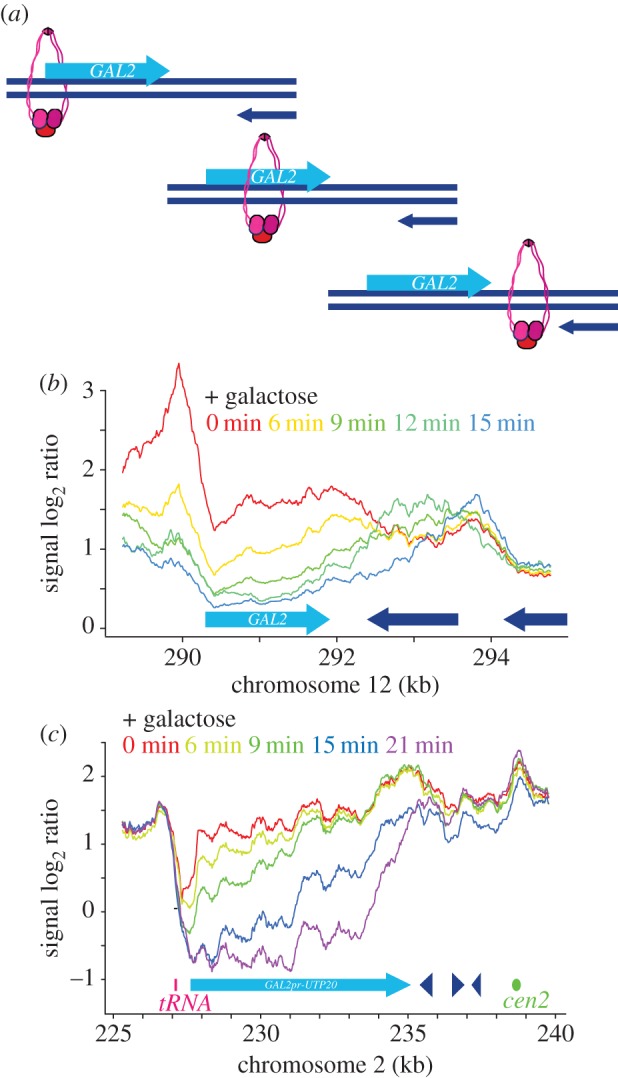
A translocation intermediate suggestive of cohesin sliding. (*a*) A model depicting a cohesin sliding intermediate. (*b*) Time course analysis of cohesin translocation at the *GAL2* locus in cells arrested in G2/M and shifted to 16°C for 1 h before galactose addition. (*c*) Cohesin behaviour at the synthetic *GAL2* promoter*-UTP20* locus, following galactose induction as in (*b*).

Our above results suggest that pre-existing chromosomal cohesin is repositioned during cohesin translocation. To observe the process at a greater time resolution, we returned to the *GAL2* locus (figure 4*a*). We again used synchronized cells that were arrested in G2/M by nocodazole treatment. To enhance the time resolution of our observations, we lowered the temperature of the culture to 16°C before *GAL2* induction by galactose addition. Samples were now collected every 3 min and processed for ChIP analysis. Cohesin along the *GAL2* locus decreased sequentially, starting 6 min after galactose addition at the 5′ end of the gene, over time progressing towards the 3′ end (figure 4*b*). At 9–12 min after galactose addition, a transient intermediate peak formed that continued to move further downstream to reach its final position 15 min after induction. These observations are consistent with a sliding movement of cohesin along chromosomes, being pushed downstream while retaining topological contact with DNA.

### 2.6. Apparent cohesin loss following transcriptional activation close to a centromere

With an aim to improve the spatial resolution when visualizing cohesin translocation, we engineered a synthetic inducible locus, making use of the 7.5 kb long *UTP20* gene, the 13th longest gene in the budding yeast genome. *UTP20* lies close to the centromere on chromosome 2, within the cohesin-enriched region that surrounds budding yeast centromeres [34]. We replaced the *UTP20* promoter with the *GAL2* promoter, so that we could switch *UTP20* expression off and on at will. The *UTP20* gene product is essential, with a role in 18S rRNA maturation [35]. Following *GAL2-UTP20* repression, cells continued to proliferate for approximately three divisions without notable adverse effect, before proliferation slowed and eventually terminated. Therefore, cultures were grown in galactose-containing medium to maintain *UTP20* expression, then shifted to medium containing raffinose just before cell synchronization by α-factor block and release. After release, cells were arrested in G2/M by nocodazole treatment, and the temperature reduced to 16°C. Galactose was added, and the cohesin distribution observed in 3 min intervals. Starting at 6 min after galactose addition, cohesin levels at the 5′ end of *UTP20* declined, after which cohesin loss spread in the 5′ to 3′ direction over time (figure 4*c*). Unlike at the *GAL2* locus, abundant cohesin was present downstream of *UTP20* already at the start of the experiment, and we did not observe further cohesin accumulation there. Instead, it appeared as if cohesin was sequentially cleared from the *UTP20* locus in the direction of transcription. Cohesin might be dislodged from chromosomes as the consequence of transcription at this locus. Alternatively, we cannot exclude that cohesin was in fact pushed downstream, but entered a more dynamic cohesin pool in the vicinity of the centromere that might have obscured the arrival of new cohesin. In either case, our findings confirm that cohesin is cleared from genes by transcription. It also reconciles our results with previous observations that, at certain loci, cohesin is apparently lost from chromosomes following transcriptional activation [27].

### 2.7. Cohesin translocation along unreplicated DNA

**Figure 5.**
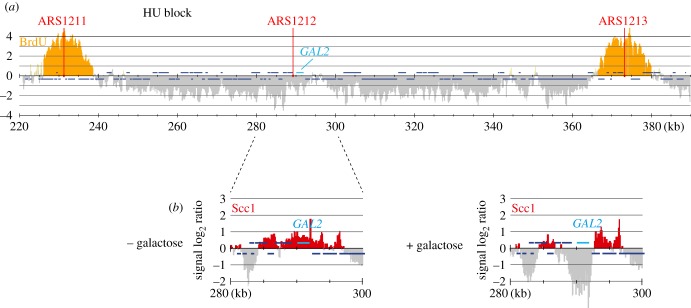
Cohesin translocation along unreplicated DNA. (*a*) Cells were synchronized in G1 and released into medium containing BrdU and 200 mM HU for 1 h. BrdU incorporation around early replicating origins was visualized by ChIP against BrdU [14]. (*b*) Cohesin translocation following *GAL2* induction in similarly synchronized and arrested cells. Scc1 association with chromatin was analysed before (− galactose) and one hour following induction (+ galactose).

So far, all our cohesin translocation experiments were performed in G2/M arrested cells in which cohesin is engaged in holding sister chromatids together. We wondered whether the ability to slide along chromosomes is a feature that cohesin gains during the establishment of sister chromatid cohesion, or whether chromosome-bound cohesin can do so already before DNA replication. To address this, we arrested cells in early S phase using HU treatment. We used a strain engineered to take up bromodeoxyuridine (BrdU) and performed ChIP against BrdU to assess which parts of the genome were replicated in this arrest. This analysis revealed BrdU incorporation at regions surrounding the early origins ars1211 and ars1213, 60 kb upstream and 80 kb downstream of the *GAL2* locus, respectively (figure 5*a*). The *GAL2* locus itself remained unreplicated in the arrest. When we induced *GAL2* transcription by galactose addition in HU-arrested cells, we observed cohesin translocation in a similar manner to what is seen in G2/M arrested cells (figure 5*b*). Thus, cohesin is able to move along chromosomes both on unreplicated and replicated DNA. This suggests that the mode of cohesin binding to DNA that endows its ability to translocate, most likely topological DNA embrace, is gained already before S phase and remains unaltered during the establishment of sister chromatid cohesion.

### 2.8. The cohesin translocation pattern is shaped by transcription termination

**Figure 6.**
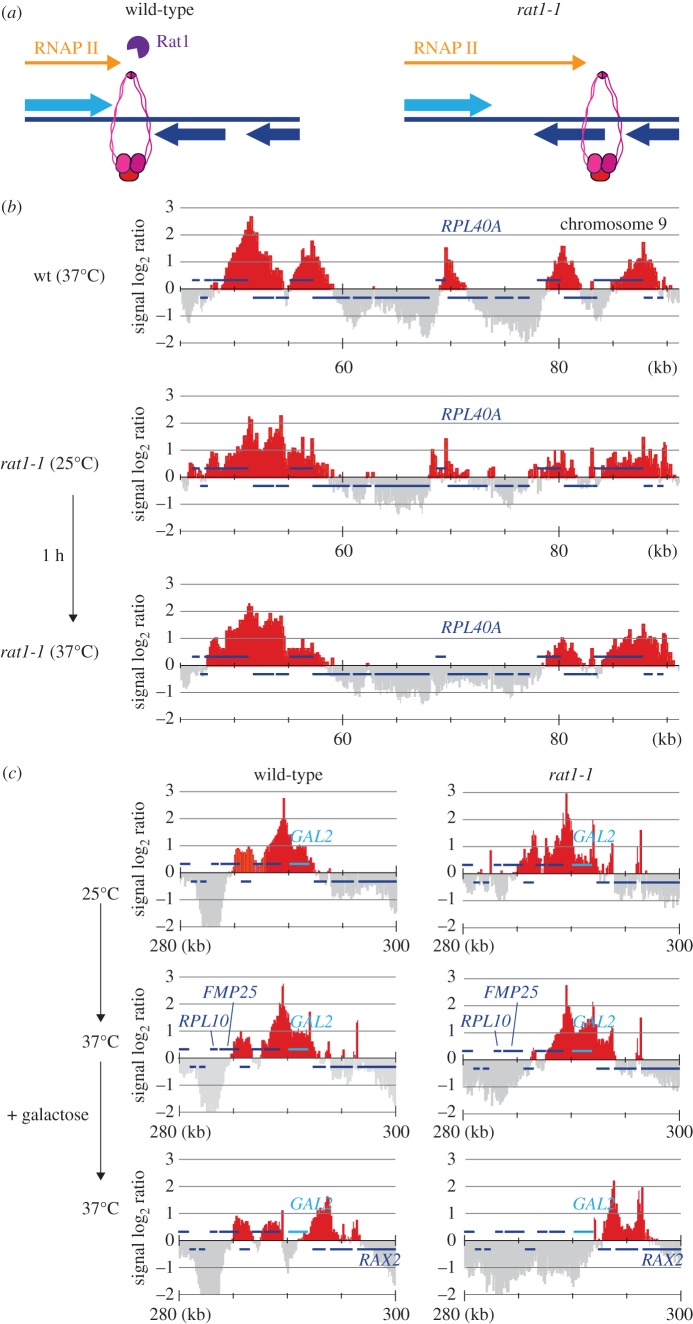
The cohesin distribution is determined by transcription termination. (*a*) A schematic of how the Rat1 nuclease aids transcription termination and of how its absence might affect cohesin positioning. (*b*) Cohesin distribution surrounding the *RPL40A* gene in G2/M arrested cells in a *rat1-1* strain and a wild-type control under the indicated conditions. (*c*) As (*b*), but the region surrounding the *GAL2* gene is shown, before the temperature shift and before and after galactose addition for 1 h.

Scc2–Scc4 cohesin loader binding sites are found in the promoters of strongly expressed genes. Cohesin can be transiently detected at these loading sites in G1 before the cohesin distribution becomes dominated by its more permanent places of residence at convergent transcriptional termination sites [7,24,30]. This pattern could be explained if most cohesin moves along chromosomes after loading, in response to transcription, until reaching places where transcription converges. To test whether the chromosomal cohesin distribution is defined by transcriptional termination, we used the temperature-sensitive *rat1-1* mutation in the Rat1 exonuclease, required for efficient transcriptional termination in budding yeast. At a non-permissive temperature for the *rat1-1* allele, RNA polymerase II progresses past normal termination points at an increased frequency [36,37] (figure 6*a*). When we compared the cohesin association pattern between wild-type and *rat1-1* cells in G2/M-arrested cells, we noted that cohesin peaks were broader and less well defined in *rat1-1* mutant cells already at the permissive temperature (figure 6*b*). Following shift to the restrictive temperature, numerous cohesin peaks disappeared, exemplified at the strongly expressed *RPL40A* gene, where the downstream cohesin peak was lost as the consequence of compromised transcriptional termination (figure 6*b*). Genome-wide, of a total of 981 prominent cohesin peaks that we counted, 167 disappeared following *rat1-1* inactivation. Additional examples from different chromosomes are shown in the electronic supplementary material, figure S1.

At the *GAL2* locus, the cohesin distribution was also broader in *rat1-1* cells at permissive temperature, compared with the wild-type control. Following shift to the restrictive temperature, the cohesin peak downstream of *RPL10* and *FMP25* disappeared. Furthermore, in response to *GAL2* induction, cohesin translocation reached further downstream with an increased proportion of cohesin accumulating as far downstream as the *RAX2* gene (figure 6*c*). These observations are consistent with the possibility that cohesin moves downstream along genes, until reaching places where transcription terminates. This further suggests that much of chromosomal cohesin reaches its final distribution by moving from promoter loading sites towards places of convergent transcriptional termination.

### 2.9. Cohesin translocation independent of gene looping

**Figure 7.**
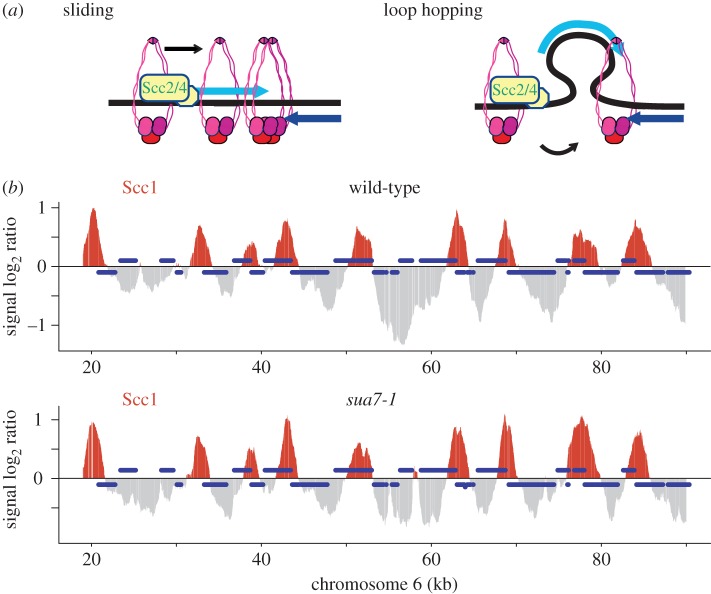
Cohesin translocation independent of gene looping. (*a*) Schematic of cohesin translocation either by sliding, or by hopping between promoter and terminator, brought together by gene looping. (*b*) Scc1 association with chromatin was compared between *sua7-1* and wild-type cells, grown at 30°C and synchronized in G2/M using nocodazole treatment. The enrichment of DNA in the Scc1 chromatin immunoprecipitate relative to a whole genome DNA sample is shown along a section of chromosome 6. See figure 1 for further details.

Gene loops have been proposed to bring gene promoters and terminators into spatial proximity in budding yeast [38]. An alternative possibility for cohesin translocation from promoters to terminators could therefore be envisioned, in which cohesin hops across loops from promoter to terminator, instead of sliding along the length of the gene (figure 7*a*). While loop hopping should not lead to translocation intermediates of the sort that we observed along the *GAL2* locus (figure 4*b*), we nevertheless wanted to investigate whether gene looping affects the chromosomal cohesin pattern. We took advantage of the TFIIB mutant *sua7-1*, which is defective in gene looping [39]. The cohesin localization pattern remained virtually unchanged between wild-type and *sua7-1* cells, with most of the cohesin concentrated at convergent transcriptional termination sites (figure 7*b*). This demonstrates that gene looping is not required for cohesin to reach terminators. Despite a largely identical cohesin pattern, we observed small differences in *sua7-1* cells, e.g. a minor new cohesin peak at ‘58 kb’ along chromosome 6 (figure 7*b*). We do not currently know whether this difference is due to the absence of gene looping, or whether it is caused by another difference in transcriptional dynamics in *sua7-1* cells [39].

### 2.10. Cohesin translocation in response to T7 RNA polymerase transcription

**Figure 8.**
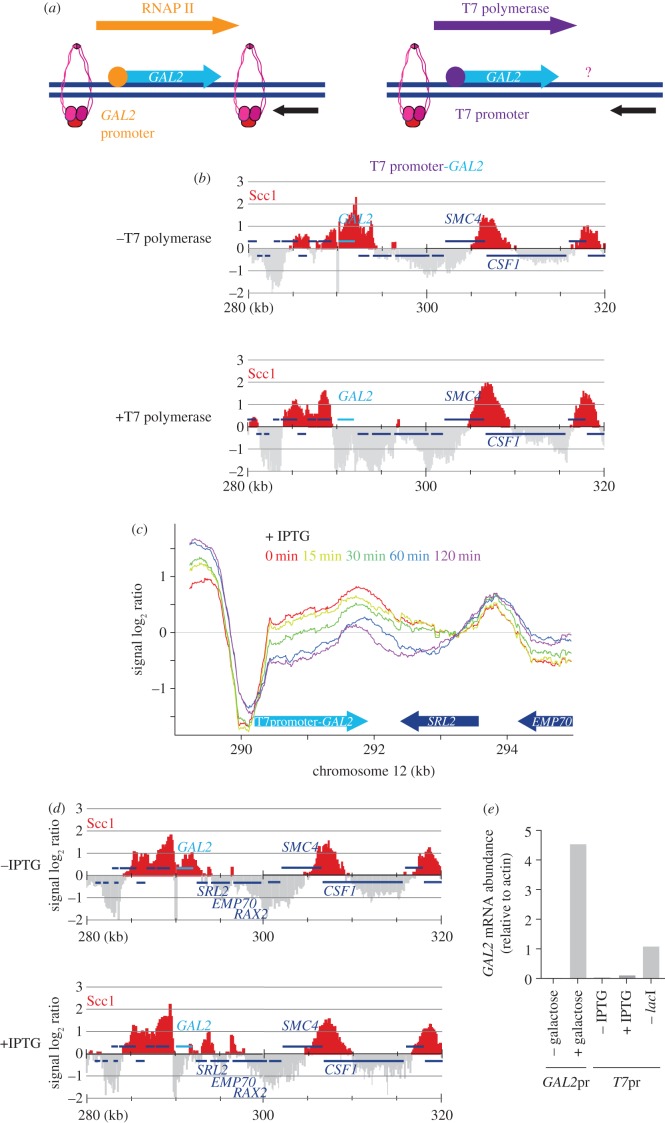
Cohesin sliding in response to T7 RNA polymerase transcription. (*a*) Schematic of how the *GAL2* gene was turned into a bacteriophage T7 RNA polymerase-transcribed locus. (*b*) Cohesin distribution at the T7 promoter-*GAL2* locus in strains that either do not (− T7 polymerase) or do (+ T7 polymerase) express a T7 RNA polymerase-NLS fusion protein. (*c*) Timecourse analysis of the cohesin distribution along the T7 promoter-*GAL2* locus, following 20 mM IPTG addition in a strain that expresses the *lac*I repressor together with the T7 RNA polymerase. (*d*) Cohesin distribution as in (*c*), following overnight culture in the absence or presence of IPTG. (e) Comparison of *GAL2* transcript levels in wild-type cells, grown overnight under conditions in which *GAL2* expression was repressed or induced, and T7 promoter-*GAL2* cells that were grown under the indicated conditions.

We finally wanted to address what aspect of transcription makes cohesin slide along chromosomes. It could be that a particular component of the RNA polymerase II machinery contacts cohesin, or simply that the sheer overall size of the greater than 2 MDa polymerase holocomplex pushes cohesin [40]. Alternatively, the RNA transcript that emerges from and moves along the chromosome with the advancing polymerase might drag cohesin along. To address this question, we engineered the endogenous *GAL2* locus such that it can be transcribed by the bacteriophage T7 RNA polymerase [41,42] (figure 8*a*). The T7 RNA polymerase is a 99 kDa monomeric protein, much smaller than the eukaryotic RNA polymerase II complex. For this purpose, we replaced the endogenous *GAL2* promoter by a T7 promoter consensus sequence and expressed the T7 RNA polymerase, fused to a nuclear localization signal, under control of the yeast housekeeping *ADH1* promoter. The cohesin distribution in cells harbouring the T7 promoter-*GAL2* locus, but lacking T7 RNA polymerase, was indistinguishable from what is observed in wild-type cells (figure 8*b*). Addition of T7 RNA polymerase resulted in striking clearance of the *GAL2* locus and downstream sequences, with hardly any cohesin detectable up to the *SMC4*-*CSF1* convergence site 15 kb further along the chromosome. This suggests that transcription by the T7 RNA polymerase efficiently removes cohesin.

We next wanted to establish whether T7 RNA polymerase pushes cohesin along chromosomes, similar to yeast RNA polymerase II, or displaces cohesin from chromosomes. Therefore, we rendered T7 transcription inducible by co-expressing the *Lac*I transcriptional repressor that binds to a *lac* operator sequence that we included as part of the T7 promoter. Cohesin distribution in the presence of both T7 RNA polymerase and *Lac*I was again comparable to that in the absence of both proteins, indicating that *Lac*I efficiently repressed *GAL2* transcription (figure 8*c*–*e*). We now added isopropyl β-d-1-thiogalactopyranoside (IPTG) to the culture to relieve *Lac*I repression and induce T7 RNA polymerase transcription. Over the course of 2 h, this led to successive clearance of the *GAL2* locus with concomitant accumulation of cohesin at the terminator of the opposing *EMP70* gene (figure 8*c*). After overnight growth in medium containing IPTG, cohesin was also detected further downstream at the *RAX2* terminator (figure 8*d*). The transcriptional strength of the induced T7 promoter in the presence of *Lac*I and IPTG was lower than if *Lac*I was altogether absent (figure 8*e*), which could be the reasons for the less complete clearance of cohesin downstream of *GAL2*. Together, these results suggest that transcription even by the relatively small T7 RNA polymerase leads to downstream translocation of cohesin along chromosomes towards places where transcription converges. While we cannot exclude direct interactions between cohesin and cellular RNA polymerases, a driving force that is sufficient for cohesin movement is provided by a feature in common with T7 RNA polymerase, possibly the RNA transcript that emerges from the polymerase.

## 3. Discussion

In this study, we analyse the nature of the striking cohesin pattern changes along chromosomes that result from transcriptional alterations. We find that factors with known roles in dynamic cohesin loading and turnover on DNA, such as the Scc2–Scc4 cohesin loader, the cohesin ATPase or Wapl, have little or no impact on cohesin movement in response to transcriptional changes. While we cannot exclude that an as-yet-unidentified mechanism unloads and re-loads cohesin during transcription, we did not find evidence that cohesin repositioning involves recruitment of new cohesin. Rather, pre-existing cohesin appears to slide along the chromosome to reach new places of residence, defined by transcription termination. These observations support a model in which cohesin that is topologically bound to DNA is able to slide laterally to adapt to the needs of other aspects of chromosome biology including, but probably not limited to, transcription.

A previous study concluded that cohesin is displaced from chromosomes by transcription [27]. While at first sight this contradicts our conclusions, on closer inspection the previous results differ only slightly from our current observations. At most of the investigated loci, both studies, as well as our previous study [24], have seen cohesin translocation in response to transcriptional activation. We do not know why at a subset of loci, e.g. the *MET17* locus, translocation is more obvious in this study than previously seen. The greater resolution of our oligonucleotide tiling microarrays, compared with a relatively sparse probe distribution [27], could be a contributing factor. At the same time, we imagine that transcription could lead to loss of cohesin at certain loci. This could be the case at places where an equilibrium between bound and free cohesin is more actively maintained, e.g. close to the centromere [34]. For the purpose of this study, we focused on analysing cohesin behaviour at places where cohesin translocation is clearly observed. Given that most cohesin loading sites in the budding yeast genome are at promoters of active genes, while most lasting places of cohesin residence correspond to areas of convergent transcriptional termination [24,28,30], we consider it likely that most of the cohesin reaches its final destination by sliding along chromosomes.

Cohesin is topologically loaded onto DNA by the cohesin loader and holds together sister chromatids by topological embrace [8,43]. If cohesin retains contact with DNA during its translocation, as suggested by our results, it implies that cohesin rings indeed slide along chromosomes. The inner diameter of cohesin of close to 35 nm [5] should enable cohesin to move along a duplex DNA, even if it is packed into 10 nm nucleosome particles and even if cohesin encircles two sister chromatids. Electrostatic interactions of the cohesin complex with DNA [8,18] are likely to add a certain friction to the movement. Thus, an active driving force is undoubtedly required to achieve translocation. This force probably stems from the movement of RNA polymerases along the DNA. The features of the transcription apparatus that lead to translocation are surprisingly generic. They are shared by the yeast RNA polymerase II holocomplex that is responsible for transcription of much of the budding yeast genome, with the evolutionarily distant T7 bacteriophage RNA polymerase. In common between these distant polymerases is the RNA transcript that emerges from them. It could be the sheer size and steric properties of the RNA transcript that drags cohesin along. We cannot exclude that endogeneous RNA polymerase machineries employ more specific ways to move cohesin. An open question of outstanding importance is why cohesin readily moves along chromosomes in response to transcription, but remains stationary during DNA replication [3,12]. Given the prevalence of cohesin translocation in response to transcription, it is tempting to speculate that a specific mechanism is in place to prevent cohesin from sliding away during replication.

In most organisms that have been studied, cohesin loader binding sites are distinct from where much of the cohesin accumulates. This includes fission yeast and mammals [44–46]. In mammalian cells, akin to what is seen in budding yeast, the cohesin loader subunit Nipped B is found in promoter regions of active genes. In budding yeast, the promoter specificity is achieved by the nucleosome landscape and the RSC chromatin remodelling complex [30]. The identifying features in mammalian cells remain to be fully understood. The mediator complex and the AP1 and other transcription factors have been implicated, but causal relationships remain to be ascertained [45,46]. After being loaded, mammalian cohesin accumulates at CTCF binding sites [47,48]. How it arrives there from its promoter loading sites is not known. If transcription moves cohesin along chromosomes in human cells, then we might expect to see a broad cohesin distribution along genes. Human genes are far longer than those found in yeasts. At a transcription elongation speed of a few kb per minute and considering that most cohesin resides on chromosomes for less than 25 min [13,49], cohesin would seldom reach a gene's 3′ end. A reported, albeit weak, cohesin enrichment upstream and downstream of genes is consistent with this scenario [48]. Notably, when acetylated and therefore more stably chromosome-bound cohesin is analysed, a marked shift to the 3′ end of short genes is observed, reminiscent of what is found in yeast [50]. In *Drosophila*, cohesin and its loader colocalize with each other, both at promoters of expressed genes and along their gene bodies [51]. Such a distribution could be explained if *Drosophila* cohesin and its loader engage in a somewhat tighter complex, compared with what is observed in other organisms. In this way, cohesin and its loader could stay associated during and after the loading reaction while cohesin moves along the gene body.

Do SMC complexes other than the cohesin complex also slide along chromosomes? In analogy to cohesin, the budding yeast condensin complex has been demonstrated to bind chromosomes by topological embrace [52]. While most of budding yeast condensin is found close to its chromosomal loading sites, that coincide with those of the cohesin complex, short-range movement of this dynamically chromosome-bound complex in response to both RNA polymerase I and II transcription has been observed [53,54]. Fission yeast condensin in turn is found in a bimodal distribution along expressed genes, enriched at both promoters and terminators [55,56], consistent with sliding along genes following loading at promoters. A third SMC complex in all eukaryotes, the Smc5/6 complex, shares many of its localization features with cohesin, including prominent enrichment at centromeres and sites of convergent transcription termination [57,58]. We therefore suggest that sliding along chromosomes is a universal feature of SMC complexes, by which these ring-shaped protein complexes stably hold onto DNA while not obstructing other chromosomal activities that have to occur at the same time.

## 4. Material and methods

### 4.1. Yeast strains and culture

All yeast strains were of the *Saccharomyces cerevisiae* W303 background, apart from the *rat1-1* mutant strain and its control, which were of FY23 background. The strain genotype details can be found in the electronic supplementary material, table S1. Cells were cultured in rich YP medium containing 2% glucose, 2% raffinose or 2% raffinose + 2% galactose as the carbon source at 25°C, or at the indicated respective restrictive temperatures for the various temperature-sensitive alleles. For amino acid starvation, cells were filtered, washed and transferred to synthetic complete SC medium lacking amino acids [59]. Cell synchronization was in all cases achieved by first arresting cells in G1 by pheromone α-factor treatment. Following release from the α-factor arrest, cells passed through a synchronous cell cycle and were arrested in early S phase or G2/M by inclusion of 200 mM HU or 5 µg ml^−1^ nocodazole in the growth medium, respectively. Cell cycle progression and cell synchrony were in all cases confirmed by FACS analysis of cellular DNA content.

### 4.2. Chromatin immunoprecipitation

At the indicated times, aliquots of the cultures were harvested and processed for ChIP of the cohesin complex as described [24]. Antibodies used for ChIP were α-Pk (clone SV5-Pk1, AbD Serotec), α-myc (clone 9E10) and α-HA (clone 12CA5). Processed chromatin immunoprecipitates and input DNA samples were hybridized to Affymetrix GeneChip *S. cerevisiae* Tiling 1.0R arrays. Presented is the genome-normalized ratio of the hybridization signals of the chromatin immunoprecipitate over the input DNA. Each bar in the bar graphs represents the average of 25 oligonucleotide probes within neighbouring 125 bp windows. For the line graphs, a smoothed moving average of 200–500 bp is shown. The microarray data are available from the GEO database under the accession number GSE80464. The quantitative analysis of cohesin binding to individual loci was performed as described, using previously described qPCR primer pairs [30], as well as primer pairs flanking the *GAL2* locus, listed in the electronic supplementary material, table S2.

### 4.3. Gene expression analysis

Cells were collected by centrifugation and total RNA was extracted using RNeasy reagents (Qiagen), following the manufacturer's instructions. Double-stranded cDNA (dscDNA) was synthesized from 10 µg of RNA using SuperScript II reverse transcriptase (Invitrogen), using random primers. Quantitative analysis of cDNA levels was then performed using a SYBR Green real-time PCR master mix (Life Technologies) and a ViiA 7 real-time PCR system (Thermo Fisher). The sequences of the primer pairs used are listed in electronic supplementary material, table S3.

## Supplementary Material

Supplementary Material
