# Supplementary material for: Evidence for cohesin sliding along budding yeast chromosomes
Source: Open Biol. 2016 Jun 8;6(6):150178. doi: 10.1098/rsob.150178 (PMC4929932; doi:10.1098/rsob.150178)
Supplement: Supplementary Material [file rsob150178supp1.pdf]

Evidence for cohesin sliding along budding yeast chromosomes

Maria Ocampo-Hafalla, Sofía Muñoz, Catarina P. Samora and Frank Uhlmann

Supplementary Material

	Page
Supplementary figure S1	2
Supplementary figure legend	3
Supplementary table S1	4
Supplementary table S2	5
Supplementary table S3	5

Figure S1

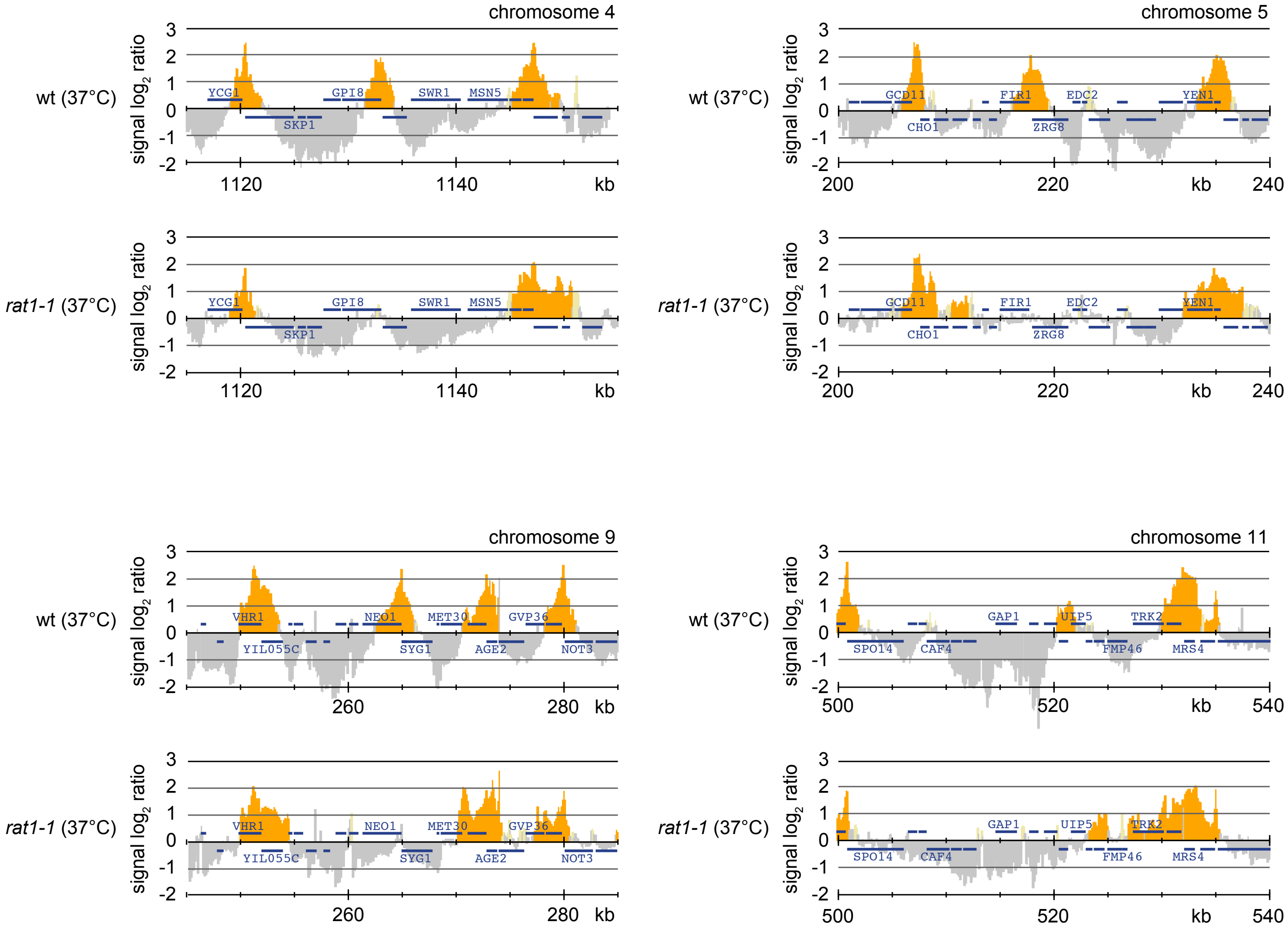
chromosome 4
chromosome 5
chromosome 9
chromosome 11
wt (37°C)
rat1-1 (37°C)
signal log2 ratio
YCG1
SKP1
GPI8
SWR1
MSN5
GCD11
CHO1
FIR1
ZRG8
EDC2
YEN1
VHR1
YIL055C
NEO1
SYG1
MET30
AGE2
GVP36
NOT3
SPO14
CAF4
GAP1
UIP5
FMP46
TRK2
MRS4
1120
1140
200
220
240
260
280
500
520
540
kb

Supplementary figure legend

Figure S1. Additional examples for cohesin pattern changes following *rat1-1* inactivation. As figure 6*b*, but cohesin association along the indicated regions from chromosomes 4, 5, 9 or 11 is compared between wild type and *rat1-1* cells at 37 °C.

Supplementary table S1. Yeast strains used in this study.

Y2269:	*MAT***a** *SCC1-Pk*$_9$*::TRP1*
Y2270:	*MAT***a** *scc2-4, SCC1-Pk*$_9$*::TRP1*
Y2342:	*MAT***a** *scc4Δ::HIS3, LEU2::scc4-4, SCC1-Pk*$_9$*::TRP1*
Y2329:	*MAT***a** *SCC1-Pk*$_3$*::HIS3*
Y2330:	*MAT***a** *smc1R58A::LEU2, smc3R58A::TRP1, SCC1-Pk*$_3$*::HIS3*
TR96:	*MAT***a** *SCC1-myc*$_9$*::TRP1*
Y3398:	*MAT***a** *wpl1Δ::kanR, SCC1-myc*$_9$*::TRP1, LEU2::tetR-GFP, URA3::TetOs*
Y2279:	*MAT***a** *scc2-4, SCC1-Pk*$_9$*::TRP1, LEU2::GAL1* promoter-*SCC1-HA*$_3$
Y4843	*MAT***a** *SCC1-HA*$_3$*::LEU2*
Y875:	*MAT***a** *GAL2* promoter-*UTP20, SCC1-Pk*$_9$*::TRP1*
Y2271:	*MAT***a** *SCC1-Pk*$_9$*::TRP1*
Y2272:	*MAT***a** *rat1-1 SCC1-Pk*$_9$*::TRP1*
Y3545:	*MAT*α *cyc1-5000 cyc7-67 cyh2 SCC1-Pk*$_3$*::LEU2*
Y3546:	*MAT*α *sua7-1 cyc1-5000 cyc7-67 cyh2 SCC1-Pk*$_3$*::LEU2*
Y3869:	*MAT***a** T7 promoter-*GAL2, SCC1-PK*$_3$*::HIS3*
Y3904:	*MAT***a** T7 promoter-*GAL2*, *SCC1-PK*$_3$*::HIS3*, *ADH1* promoter-*NLS-T7 polymerase::TRP1*
Y4883:	*MAT***a** T7 promoter-*GAL2*, *SCC1-PK*$_3$*::HIS3*, *ADH1* promoter-*NLS-T7 polymerase::TRP1, ADH1* promoter-*NLS-LacI::LEU2*

Supplementary table S2. qPCR primer pairs used for quantitative analysis of Scc1 chromatin immunoprecipitates at the *GAL2* locus.

Name	Sequence
*GAL2*F-2 (P1)	TTGGCCTGGATGATTCCT
*GAL2*R-2 (P1)	AGCGCCCAAAAGTAAACA
3'RegionF-2 (P2)	CTTGCTTACTTATCCACTCCCA
3'RegionR-2 (P2)	TGTACAGGGTTAATCTATAGCCACA

Supplementary table S3. qPCR primer pairs used for gene expression analysis.

Name	Sequence
*GAL2*F (SM88)	CGAACTCAGTTCAATGGAGAGT
*GAL2*R (SM89)	TACCGGCCATGATCAGATCT
ActinF (MRT1)	CTCCACCACTGCTGAAAGAGAA
ActinR (MRT2)	CGAAGTCCAAGGCGACGTAA
